# A modified choline-deficient, ethionine-supplemented diet reduces morbidity and retains a liver progenitor cell response in mice

**DOI:** 10.1242/dmm.022020

**Published:** 2015-12-01

**Authors:** Adam M. Passman, Robyn P. Strauss, Sarah B. McSpadden, Megan L. Finch-Edmondson, Ken H. Woo, Luke A. Diepeveen, Roslyn London, Bernard A. Callus, George C. Yeoh

**Affiliations:** 1 School of Chemistry and Biochemistry, The University of Western Australia, Crawley, Western Australia 6009, Australia; 2 Cancer and Cell Biology Division, The Harry Perkins Institute of Medical Research, Nedlands, Western Australia 6009, Australia; 3 School of Health Sciences, The University of Notre Dame Australia, Fremantle, Western Australia 6959, Australia

**Keywords:** Ductular reaction, Hepatic stem cell, Hepatocarcinogenesis, Fatty liver, Regeneration

## Abstract

The choline-deficient, ethionine-supplemented (CDE) dietary model induces chronic liver damage, and stimulates liver progenitor cell (LPC)-mediated repair. Long-term CDE administration leads to hepatocellular carcinoma in rodents and lineage-tracing studies show that LPCs differentiate into functional hepatocytes in this model. The CDE diet was first modified for mice by our laboratory by separately administering choline-deficient chow and ethionine in the drinking water (CD+E diet). Although this CD+E diet is widely used, concerns with variability in weight loss, morbidity, mortality and LPC response have been raised by researchers who have adopted this model. We propose that these inconsistencies are due to differential consumption of chow and ethionine in the drinking water, and that incorporating ethionine in the choline-deficient chow, and altering the strength, will achieve better outcomes. Therefore, C57Bl/6 mice, 5 and 6 weeks of age, were fed an all-inclusive CDE diet of various strengths (67% to 100%) for 3 weeks. The LPC response was quantitated and cell lines were derived. We found that animal survival, LPC response and liver damage are correlated with CDE diet strength. The 67% and 75% CDE diet administered to mice older than 5 weeks and greater than 18 g provides a consistent and acceptable level of animal welfare and induces a substantial LPC response, permitting their isolation and establishment of cell lines. This study shows that an all-inclusive CDE diet for mice reproducibly induces an LPC response conducive to *in vivo* studies and isolation, whilst minimizing morbidity and mortality.

## Abstract (precis)

**Summary:** This modified choline-deficient, ethionine-supplemented model induces liver injury in mice and reproducibly minimizes morbidity and mortality, whilst maintaining a liver-progenitor-cell response sufficient for cell-line establishment.

## INTRODUCTION

The CDE (choline-deficient, ethionine-supplemented) diet is a physiologically relevant model of liver disease because it mimics the human condition of chronic fatty liver disease. This model mirrors other liver pathologies such as alcoholic liver disease and viral hepatitis, in which inflammation is invariably present and cells known as LPCs (liver progenitor cells), or hepatic progenitor cells, are observed (Libbrecht and Roskams, 2002). Short-term application results in steatosis, inflammation and LPC proliferation, followed by fibrosis, cirrhosis and hepatocellular carcinoma (HCC) in long-term studies (Knight et al., 2000; Davies et al., 2006; Ochoa-Callejero et al., 2013). The time to development of HCC in mice fed this diet is protracted, thereby mimicking the extended development period of HCC in humans.

The degree to which LPCs contribute to liver regeneration remains contentious. Recently, a range of lineage-tracing strategies and dietary models (including the CDE diet) have been used to assess whether LPCs differentiate *in vivo* into hepatocytes. Some studies (Rodrigo-Torres et al., 2014; Schaub et al., 2014; Tarlow et al., 2014a,b; Yanger et al., 2014) have concluded that there is little evidence to support LPCs as a source of hepatocytes. Others report that the CDE diet is the only liver-injury model in which LPCs differentiate into substantial numbers of hepatocytes, thereby contributing to hepatic regeneration (Espanol-Suner et al., 2012; Affo and Sancho-Bru, 2014; Shin et al., 2015). These conflicting results exemplify the need for a defined, reproducible CDE dietary protocol. The CDE diet was originally used in rats (Shinozuka et al., 1978; Shin et al., 2015), and it was later modified for mice by our laboratory (Akhurst et al., 2001). This modification involved administering choline-deficient chow with ethionine-supplementation in drinking water and was designated the CD+E diet.

The decade since this publication has seen an increase in awareness regarding ethical animal research and commensurate tightening of regulatory compliance guidelines. Our protocol (Akhurst et al., 2001) no longer satisfied our institution's guidelines. Our laboratory has also received numerous communications from researchers experiencing difficulties using this protocol. Major concerns included body weight loss in excess of 20% and morbidity (including hunching caused by abdominal pain, and decreased movement and grooming). In many instances, welfare concerns resulted in premature culling necessitating increased animal usage in anticipation of animal loss. Apart from ethical concerns, there was substantial animal-to-animal variation in the LPC response.

Akhurst et al. uncoupled the choline deficiency from the ethionine supplementation so that each component could be altered independently to better suit different strains of mice and the duration of treatment (Akhurst et al., 2001). However, this approach introduces variability because chow and ethionine intake can vary between animals. Additionally, the age and weight of the mice could influence their nutritional habits. We hypothesize that this combination of factors is a major source of the adverse effects observed in some mice. We propose that varied consumption, particularly during the first week of diet administration, when mice are experiencing the most severe effects of the diet, leads to a wide range of animal conditions and LPC responses. In this study, we use an all-inclusive CDE diet with ethionine incorporated in choline-deficient chow, which ensures that the choline deficiency is linked with ethionine supplementation. We varied the strength of the diet by mixing it with control chow. These were tested with detailed recording of the initial age and weight of mice, to establish a protocol that achieves an optimal balance between the LPC response and animal welfare, characterized by low morbidity, acceptable weight loss and high survival rates.

**RESOURCE IMPACT**

**Background**

The choline-deficient, ethionine-supplemented (CDE) diet is a well-established tool for studying liver disease because it induces a liver progenitor cell (LPC) response (to repair damaged liver) and closely recapitulates disease progression in humans. With short-term administration, the diet leads to fatty liver in rodents, and long-term administration eventually causes liver cancer. Originally used in rats, the Yeoh laboratory adapted and modified this diet for application in mice. This modification involved administering choline-deficient chow with ethionine supplementation in drinking water and was designated the CD+E diet. Since that time, there has been an increased awareness of ethics in animal research and, given that the diet can result in body weight loss and morbidity, it has become increasingly difficult to gain approval for its use as a model. Experimentally, there has also been substantial animal-to-animal variation in the LPC response. Combined, these factors necessitated further modification of the CD+E diet for use in mice.

**Results**

In this study, the authors modified the CD+E diet to include ethionine in the chow. The resulting modification was designated the CDE diet. The CDE diet was tested, using a range of strengths, for any effects on mortality, morbidity, liver damage and LPC response. The 67% and 75% CDE diet strengths had considerably favorable morbidity and mortality outcomes compared to a 100% CDE diet. Of the 67% and 75% diets, the former was associated with a lower level of liver damage and a more subdued LPC response. Despite this, the 67% CDE diet was sufficient for the isolation of LPCs and derivation of a cell line. The cell line that was established expressed the marker proteins EpCAM, E-cadherin and CK19, and was able to differentiate into more mature hepatic cell types, thus demonstrating the hallmark characteristic of LPCs.

**Implications and future directions**

The ability of LPCs to expand and differentiate means that they have potential as a form of cellular therapy for the treatment of liver disease. However, their association with diseased liver necessitates further research to ensure their safety. The modified model reported in this study ensures the reliable continuation of the CDE diet within the context of tightening ethical guidelines. Applied at a strength of 67%, the CDE diet described here reproducibly induces an LPC response conducive to *in vivo* studies of liver disease and establishment of LPCs, whilst minimizing morbidity and mortality in test animals. This work is particularly important in light of the growing global obesity epidemic and associated liver disease, which demand the development of new models and tools for the scrutiny of underlying mechanisms and candidate therapies.

## RESULTS

### Survival depends on CDE strength, and mouse age and weight

A preliminary study was conducted using a 100% CDE diet. Mice fed the 100% CDE diet displayed considerable morbidity and were culled within 3 to 5 days on ethical grounds. Consequently, reduced strength CDE diets (67%, 70% and 75%) were trialed. In the first experiment, 5-week-old mice, ranging from 13.90 g to 17.86 g (average 15.97 g) weight, were fed CDE diets. Despite the reduced strength, many mice did not tolerate the diet, displaying signs of morbidity and body weight loss in excess of 18%, necessitating euthanasia during the first week. Survival rates for the 67% and 75% CDE diets were 71% and 43%, respectively (Table 1). The 70% CDE diet yielded the same survival outcomes as the 75% CDE diet (data not shown).

**Table 1.**
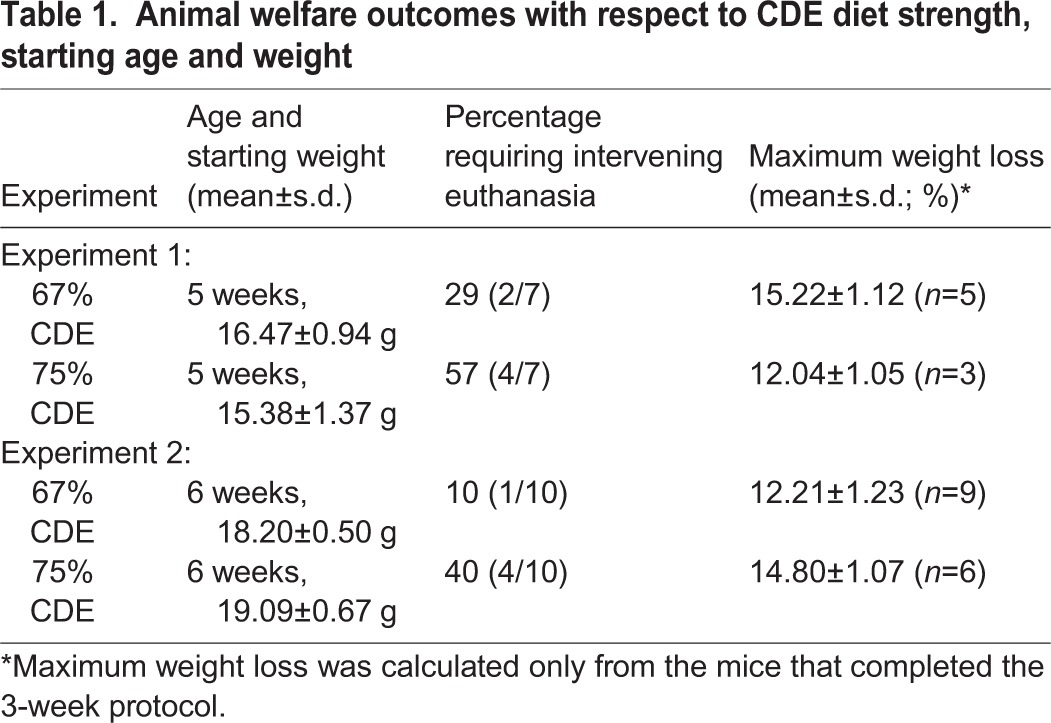
**Animal welfare outcomes with respect to CDE diet strength, starting age and weight**

Scrutiny of the data indicated that mice requiring intervening euthanasia had significantly lower starting weights (15.19 g) compared to mice that survived the 3-week experiment (16.44 g, *P*<0.001). To improve the survival rate, a second experiment utilized mice which were both older than 5 weeks and heavier (≥18 g). These were placed on only the 67% and 75% CDE diets. Survival rates increased to 90% and 60% for the 67% and 75% CDE diets, respectively (Table 1). Subsequent data presented are derived from this experiment.

### Both dietary strengths cause transient morbidity and weight loss

Animal morbidity and weight loss were monitored to determine which strength of CDE diet minimized adverse animal welfare impact. Several mice from both CDE groups displayed signs of discomfort, including slight hunching and slowed movement; other outward signs of distress were rare. Animals on the control chow never showed signs of discomfort or distress and all received a score of zero. Mice fed the 67% and 75% CDE diets had an average score of 0.17±0.10 and 0.31±0.19 points per day, respectively, although this difference was not statistically significant. Typically, mice in both CDE groups experienced the greatest morbidity scores and weight loss during the first week on the diet (Fig. S1). None of these mice accumulated a morbidity score to warrant intervening euthanasia and all preliminary culls were solely due to body weight loss. By day 10, all CDE mice had returned to control-level morbidity scores. The majority of these mice began regaining weight after day 6 and their weights were not significantly different from control mice at the conclusion of the experiment (Fig. S1).

### Liver damage increases with dietary strength

**Fig. 1.**
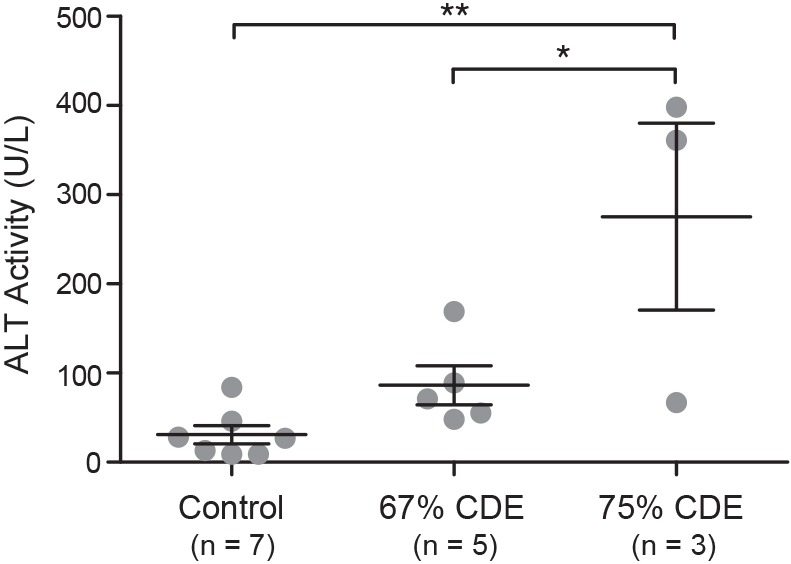
**Serum ALT activity correlates with dietary strength.** Serum ALT levels were analyzed from mice fed control, or a 67% or 75% CDE diet and are presented as mean±s.e.m. Significance was determined by one-way ANOVA (*P<0.05, **P<0.01).

After 3 weeks, serum alanine transferase (ALT) activity was significantly lower (*P*<0.05) for mice on the 67% CDE diet (86.4 U/l) compared with those on the 75% CDE diet (275.3 U/l; Fig. 1). Unlike ALT activity for mice on the 75% CDE diet (*P*<0.01), ALT activities for mice on the 67% CDE diet were not significantly different from control mice (Fig. 1). Serum γ-glutamyltransferase (GGT), a marker of biliary cell necrosis (Leonard et al., 1984), was not significantly altered in mice receiving the CDE diet (Fig. S2). The possibility of damage to other organs was also evaluated. Pancreatic damage was assessed because CDE dietary regimes have been used as models for pancreatitis (Lombardi et al., 1975; Ida et al., 2010; Wang et al., 2010). We found a statistically significant decrease in serum amylase activity in mice that received the CDE diet (Fig. S3A). Hematoxylin and eosin staining of CDE pancreas showed no evidence of inflammatory infiltrates (Fig. S3B,C). Damage to any other organ that was exposed in the abdominal and pelvic cavities was not evident upon visual inspection.

### LPC response increases with dietary strength

**Fig. 2.**
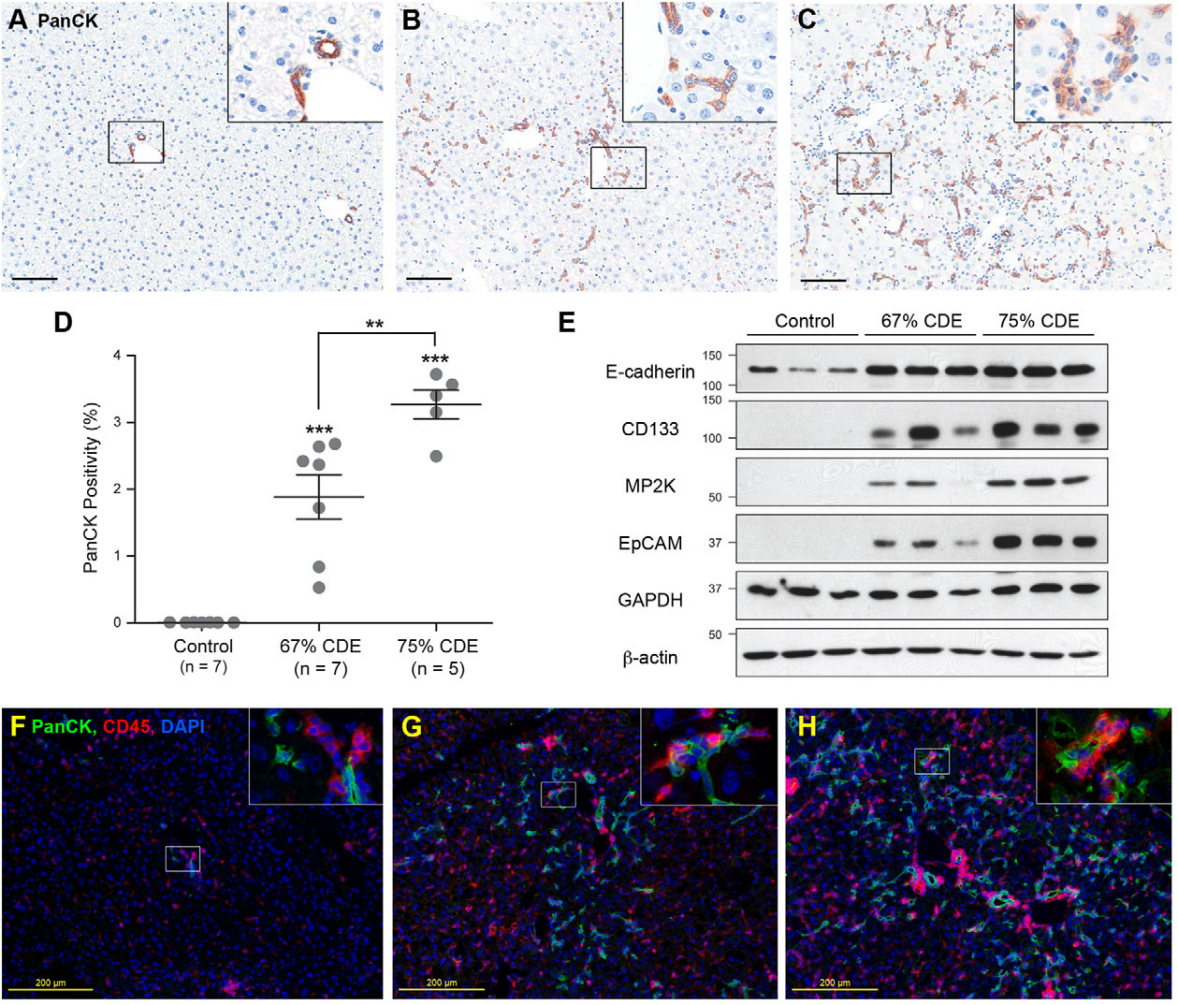
**LPC and inflammatory responses correlate with dietary strength.** Liver sections from mice fed the control (A), 67% (B) and 75% (C) CDE diets for 3 weeks were stained for PanCK. Scale bars: 100 µm. Insets are magnified threefold. (D) The percentage of PanCK^+^ pixels (PanCK positivity) was determined and is shown as mean±s.e.m. Significance was determined by ANOVA (**P<0.01, ***P<0.001). (E) Protein lysates from the livers of mice on each diet were immunoblotted for E-cadherin, CD133, M2PK and EpCAM. GAPDH and β-actin were used as loading controls. (F-H) Liver sections from mice fed the control (F), 67% (G) and 75% (H) CDE diets for 3 weeks were stained for CD45 (red), PanCK (green) and counterstained with DAPI. Insets are magnified threefold.

In the livers of control mice, pan-cytokeratin-positive (PanCK^+^) LPCs were only observed periportally (Fig. 2A). Livers from mice fed 67% (Fig. 2B) or 75% (Fig. 2C) CDE diets showed enhanced PanCK^+^ cell populations that had migrated into the parenchyma, typical of the LPC or ductular response observed in CD+E diet mice (Tirnitz-Parker et al., 2007). PanCK positivity was significantly lower (*P*<0.01) in the livers of mice on the 67% CDE diet compared to the 75% CDE diet (Fig. 2D). Both dietary regimes increased PanCK staining over 300-fold compared to controls. Abundance of LPC marker proteins E-cadherin, CD-133, M2PK and EpCAM was increased in the livers of the 67% and 75% CDE-diet mice compared to controls (Fig. 2E). As expected, CD45^+^ inflammatory cells were detected in all livers but their numbers were elevated in the 67% and 75% CDE samples compared with controls (Fig. 2F-H).

### LPCs can be established from CDE mice

**Fig. 3.**
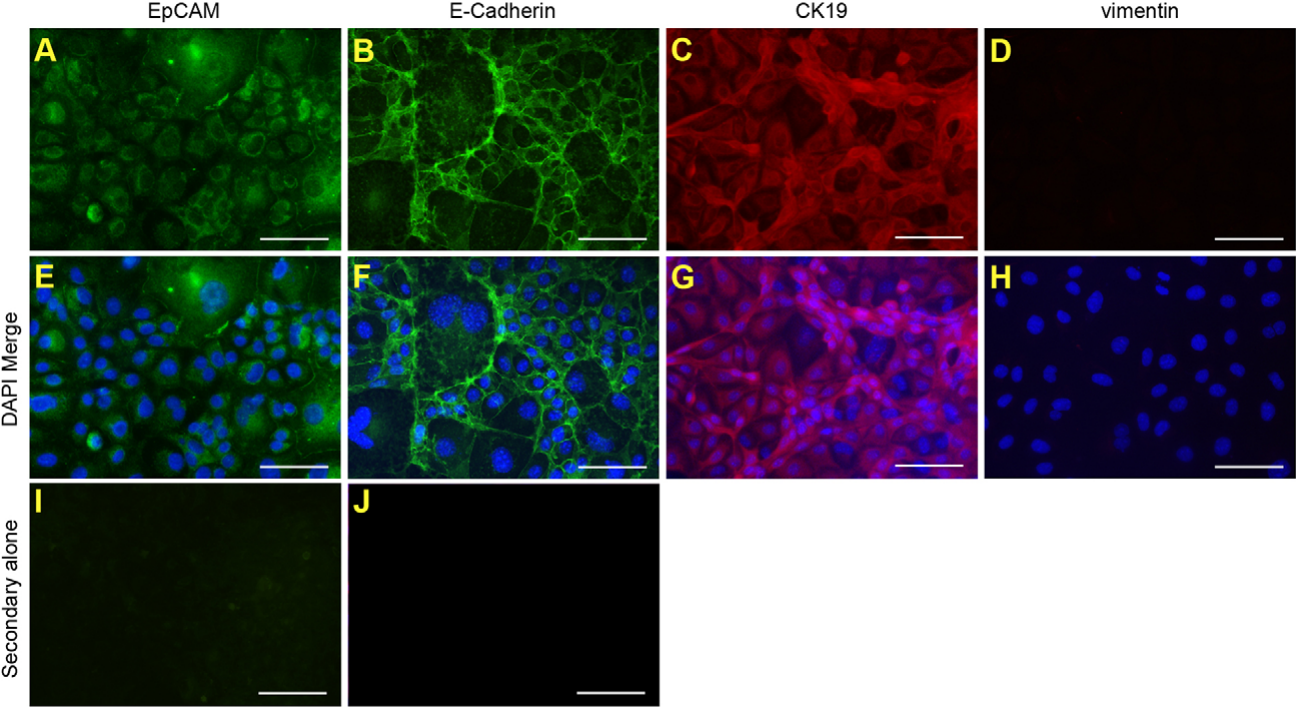
**Cell lines derived from mice fed a CDE diet express LPC markers.** The LPC line, BMOL3, was derived from a mouse fed a 67% CDE diet. Immunofluorescence was used to detect known LPC markers [EpCAM (A,E), E-cadherin (B,F) and CK19 (C,G)] and mesenchymal marker vimentin (D,H). Representative images are shown without (A-D) and with (E-H) DAPI staining. Control staining with either Alexa-Fluor-488 goat-anti-rabbit or -594 goat-anti-rat alone are shown (I,J). Scale bars: 100 µm.

**Fig. 4.**
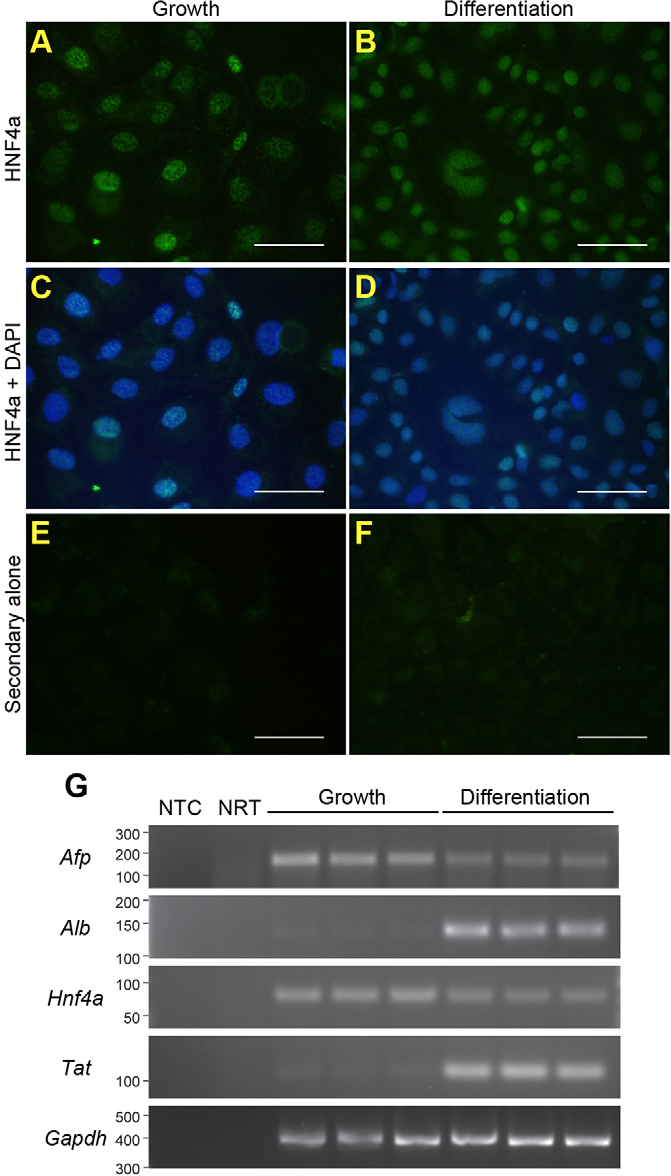
**BMOL3 LPCs can differentiate towards the hepatocyte lineage.** BMOL3 cells were treated to differentiate them towards hepatocytes. Proliferating (A,C,E) and differentiated (B,D,F) LPCs were stained for HNF4α (A,B). Images are shown without (A,B) and with (C,D) DAPI staining. Control staining with Alexa-Fluor-488 rabbit-anti-goat alone is shown (E,F). Scale bars: 50 µm. RT-PCR was used to determine abundance of the hepatocyte markers *Afp*, *Alb*, *Hnf4α* and *Tat*. *Gapdh* was used as a loading control. Non-template (NTC) and no reverse transcriptase enzyme (NRT) were included as controls.

**Fig. 5.**
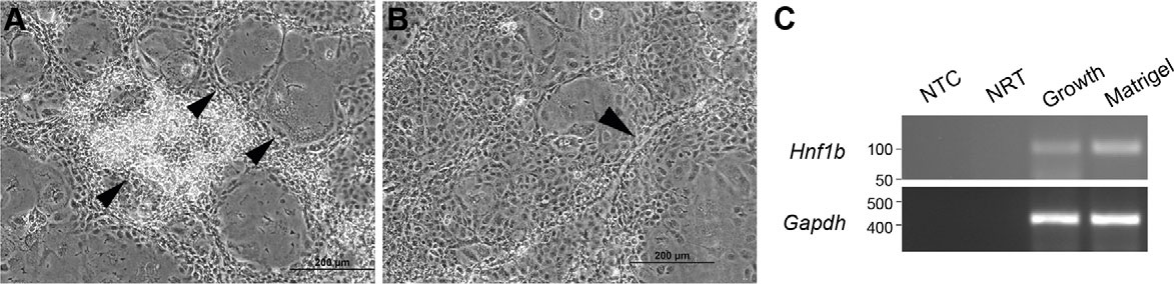
**BMOL3 LPCs can differentiate towards cholangiocytes.** The LPC line BMOL3 was grown in Matrigel™ to induce differentiation along the cholangiocyte lineage. Ductal structures were evident after 10 days as indicated by arrowheads (A,B). RT-PCR was used to confirm expression of the biliary marker *Hnf1b* in differentiated cells (C). *Gapdh* was used as a loading control. Non-template (NTC) and no reverse transcriptase enzyme (NRT) controls were also included.

Typically, the CD+E diet substantially increases LPC numbers, permitting the generation of cell lines. To verify whether LPC lines could be established from CDE-fed mice, a putative LPC line was generated from a 67% CDE-fed mouse. The cell line, bipotential murine oval liver 3 (BMOL3), expressed the LPC markers epithelial cell adhesion molecule (EpCAM), epithelial cadherin (E-cadherin) and cytokeratin 19 (CK19) (Fig. 3A-C,E-G), and did not express the mesenchymal marker vimentin (Fig. 3D,H). BMOL3's bipotentiality toward the hepatocyte and cholangiocytes lineages was confirmed. Nuclear hepatocyte nuclear factor 4 alpha (HNF4α) staining was observed in proliferating and differentiated LPCs (Fig. 4A-D). Differentiation along the hepatocyte lineage induced expression of the mature-hepatocyte markers *Alb* (albumin) and *Tat* (tyrosine aminotransferase). A commensurate decrease in *Afp* (alpha fetoprotein) transcript abundance was observed (Fig. 4E). Ductal structures (Fig. 5A,B) were detected and *Hnf1b* (hepatocyte nuclear factor 1 homeobox B) expression was upregulated (Fig. 5C) when differentiated along the cholangiocyte lineage.

## DISCUSSION

Given the stringent ethical framework governing animal experiments, we propose that the CDE protocol described here is superior to the previously described CD+E protocol (Akhurst et al., 2001). The strength of an all-inclusive CDE diet can be finely tuned by altering the ratio of CDE chow to control chow. This affords more consistent outcomes for LPC response and animal welfare. Furthermore, because the CDE components are not consumed separately as in the CD+E protocol, animals receive the same dosage of ethionine relative to choline deficiency throughout the experiment, thereby reducing animal-to-animal variation. Despite the inclusion of ethionine in the chow, there was no evidence of reduced palatability: mice in CDE groups consumed similar amounts of food per day to those in control groups (data not shown).

Of the reduced strengths tested, the 67% CDE diet resulted in greater animal survival, lower morbidity scores and less liver damage. Serum ALT activity, which served as a measure of hepatocytic damage, was not significantly elevated in the 67% CDE mice relative to controls at the conclusion of the 3-week protocol. Interestingly, ALT activity levels in the 75% CDE mice remained significantly elevated at 3 weeks. This prolonged liver damage is undoubtedly the reason that the LPC response was greater in the 75% CDE mice, and they had relatively poorer welfare outcomes. The possibility that biliary injury was responsible for the LPC response was investigated by analyzing serum GGT activity. Activities from CDE mice were not significantly different from controls. Finally, our results indicate that morbidity as a result of damage to other organs is unlikely, because all other organs appeared normal by visual inspection, no pancreatic inflammation was observed, and serum amylase was in fact significantly lower in CDE specimens. Combined, the data support the view that the LPC response and morbidity is a consequence of damage to the liver, more specifically, the hepatocytes.

In addition to dietary strength and composition, starting weight and/or age of the mice might have played a role in the success of the CDE diet. Starting mice on the CDE diet when they weighed more than 18 g reduced mortality. Age might also be a contributing factor because the heavier mice were also older. Similarly, Lombardi et al. found that young mice (4.3 weeks) have lower survival rates than adult mice (10 weeks) on the CDE diet (Lombardi and Rao, 1975). We recommend that it is important to evaluate animal welfare by applying a morbidity score that encompasses multiple parameters rather than a single measure such as weight loss to more accurately reflect the health of the animals.

Our findings lead us to recommend the 67% CDE diet for the majority of CDE experiments because this strength maximizes animal survival and induces a sufficient LPC response to permit their isolation and cell-line derivation. The 67% CDE diet might be more suited for mouse strains that are highly sensitive to the CDE diet, including the BALB/c strain (Knight et al., 2007). In a lineage-tracing study using the CDE diet, Shin et al. proposed that mice required at least 14% weight loss to achieve *in vivo* differentiation of LPCs to hepatocytes (Shin et al., 2015). Higher levels of liver damage were commensurate with the 14% weight-loss threshold, suggesting that a substantial degree of liver damage is required for LPC differentiation into hepatocytes *in vivo*. With this in mind, a 75% CDE diet might be more appropriate for lineage-tracing studies, because mice on this strength achieve ALT levels similar to those seen by Shin and colleagues (2015). Furthermore, we speculate that substantial liver damage, weight loss greater than 14%, and a substantial LPC response might be required for the development of HCC from the LPC niche. Ultimately, the choice of which diet to use will depend on the specific circumstances and the aims of the study. The all-inclusive CDE diet affords researchers the flexibility to administer a diet that consistently induces a robust LPC response whilst minimizing negative impacts on the animals.

## MATERIALS AND METHODS

### Animals and diet administration

Diluted chow (75%, 70% or 67%) was prepared by combining 100% CDE (MP Biosciences, Seven Hills, NSW, Australia, Cat. 0296021410) with control (choline-sufficient) chow (MP Biosciences, Cat. 0296041210). Chows were ground into powder and recombined as necessary using 10 ml sterile tap water per 100 g chow. Recombined chow was stored at 4°C for up to a week or at −20°C for up to a month.

C57Bl6/J male mice (4- to 5-weeks old or 14-16 g) were supplied by the Animal Resource Centre (Murdoch, WA, Australia). Mice were housed 4-5 mice per cage in filter lid cages within a temperature-controlled room with alternating 12 h light and 12 h dark cycles. Mice were fed, *ad libitum*, standard chow for 2 days then switched to control chow for 3-5 days before administration of the CDE diet for 3 weeks.

Cages and bedding (fine aspen) were changed twice a week. Chow was replaced daily and drinking water was replaced weekly. All animal procedures were approved by the University of Western Australia (UWA)'s Animal Ethics Committee and compliant with National Health and Medical Research Council (NHMRC; Australia) guidelines.

### Animal monitoring

Mice were monitored daily before CDE administration, then twice daily until mice recovered to within 5% of their CDE day-0 weight, and daily thereafter. Mice were weighed and observed. Morbidity points were allocated depending on the severity of the symptoms observed (Table S1). Weight loss was calculated based on CDE day-0 weight. According to UWA Animal Ethics Committee stipulations, a mouse was culled if deemed it would exceed 20% weight loss, or if a morbidity score of 3 or above was attained.

### Tissue and blood collection

Mice were anesthetized with Avertin (Sigma, Sydney, NSW, Australia, Cat. T48402; 600 µg/g body weight). The abdominal cavity was exposed and a perfusion needle was inserted into the hepatic portal vein. Blood was collected by cardiac puncture directly after perfusion-needle insertion. Blood was allowed to coagulate then centrifuged at 16,000 ***g*** for 10 min at 4°C to separate serum. The liver was perfused with 37°C sterile phosphate buffered saline (PBS, pH 7.4) at a rate of 5 ml/min until cleared of blood. Livers were snap-frozen for protein extraction, or fixed for 24 h at room temperature (RT) in 10% buffered formalin before embedding in paraffin.

### Serum alanine transferase (ALT), γ-glutamyltransferase (GGT) and amylase assays

ALT assays were performed commercially by PathWest Laboratory Medicine (Nedlands, Western Australia) using the Activated Alanine Aminotransferase assay (Abbott Diagnostics, North Ryde, NSW, Australia, Cat. 8L92) per the manufacturer's instructions. Serum GGT and amylase assays were performed as per the manufacturer's instructions (Sigma, Sydney, NSW, Australia, Cat. MAK090 and MAK009, respectively).

### LPC culture

LPCs were isolated from mice as outlined previously (Tirnitz-Parker et al., 2007). Cell lines were established using the ‘plate and wait’ method described previously (Strick-Marchand and Weiss, 2002). LPCs were cultured in Williams' E Medium (WEM; Sigma, W4125) containing supplements [2.5 µg/ml Fungizone (Life Technologies, Melbourne, VIC, Australia, Cat. 15290-018), 48.4 µg/ml Penicillin (Merck Millipore, Kilsyth, VIC, Australia, Cat. 5161), 675 µg/ml Streptomycin (Life Technologies, Cat. 11860-038), 2 mM Glutamine (Sigma, Cat. G8540), 5% fetal bovine serum (Life Technologies, Cat.16000-044), 20 ng/ml epidermal growth factor (In Vitro Technologies, Nobel Park North, VIC, Australia, Cat. FAL354001), 30 ng/ml insulin-like growth factor-II (GroPep Bioreagents, Thebarton, SA, Australia, Cat. OU001) and 0.25 U/ml Humulin R (Eli Lilly and Company, West Ryde, NSW, Australia supplied through the UWA Pharmacy)]. The 10:10 protocol was used to induce hepatic differentiation as described (Tirnitz-Parker et al., 2007). For cholangiocyte differentiation, LPCs were grown in Matrigel™ (In Vitro Technologies, Cat. 354234) for 10 days in WEM with supplements. Once established, cell lines were tested for mycoplasma and murine hepatitis virus contamination by PathWest, and certified pathogen-free. Cells were also confirmed to be of *Mus musculus* origin by PCR amplification of mouse-specific cytochrome c oxidase I, mitochondrial (*Cox1*; Fwd: 5′-AGTACAGCAGCGGGAGCATGC-3′, Rev: 5′-TTGAGGAGGACCGTGAAGCCG-3′).

### Immunohistochemistry and LPC quantification

Formalin-fixed sections (4 µm) were dewaxed and rehydrated. Antigen retrieval was performed with Proteinase K (Dako, North Sydney, NSW, Australia, Cat. K0690; 40 µg/ml in Tris buffered saline) for 5 min. Sections were treated with 3% H_2_O_2_ for 10 min followed by Serum-Free Protein Block (Dako, Cat. X0909). Wide-spectrum cytokeratin (PanCK; Dako, Z0622; 1:400) polyclonal antibody was applied overnight at 4°C. Staining was detected with LSAB+ (Dako, Cat. K0690) and visualized with DAB+substrate (Dako, Cat. K0690) per the manufacturer's instructions. Slides were counterstained with hematoxylin, dehydrated and mounted. Slides were scanned at 40× magnification using the Aperio Scanscope XT and software (Vista, CA, USA) for viewing and quantitation. The edges of the tissue, bile ducts and lumens were excluded from analysis. Parameters of the ‘Positive Pixel Count v9.1’ algorithm were adjusted to calculate the number of stained pixels as a percentage of total pixels within the section (positivity).

### Immunofluorescence

Frozen liver sections (7 µm) were stained with anti-PanCK and anti-CD45 (BD Pharmingen, Cat. 550539). Anti-EpCAM (Sapphire Bioscience, Waterloo, NSW, Australia, Cat. ab71916), anti-E-cadherin (Genesearch, Arundel, QLD, Australia, Cat. 3195S), anti-CK19 (TROMA III, a gift from Rolf Kemler at the Max-Planck Institute), anti-vimentin (BioScientific, Sydney, NSW, Australia, Cat. MAB2105) and anti-HNF4α (hepatocyte nuclear factor 4 alpha; Thermo Fisher Scientific, Scoresby, VIC, Cat. SCZSC-6556) antibodies were used to stain cultured LPCs. Samples were fixed in acetone-methanol (v/v, 1:1), washed in PBS, blocked in 5% bovine serum albumin (BSA) in PBS for 30 min and incubated with primary antibody (1:200 in 1% BSA/PBS) overnight at 4°C. Samples were washed and incubated with either Alexa-Fluor-488 goat anti-rabbit IgG, 594 goat-anti-rat IgG or 488 rabbit anti-goat IgG secondary antibody (Life Technologies, Cat. A11008, A11007 and A11078, respectively; 1:200 in 1% BSA/PBS) for 1 h at RT. Samples were stained with DAPI, washed and mounted with Gelvatol medium as described previously (Finch et al., 2015). Fluorescence was imaged with an IX2-ILL100 Inverted Microscope (Olympus, Macquarie Park, Australia).

### Western blotting

Tissues were homogenized before being lysed and immunoblotted as described (Finch et al., 2015). Antibodies used were: anti-E-cadherin (Genesearch; 1:5000), anti-EpCAM (Sapphire Biosciences; 1:1000), anti-M2PK (Genesearch, Cat. 3198S; 1:1000), anti-CD133 (Sapphire Bioscience, Cat. PAB12663; 1:1000), anti-β-actin (Sigma, Cat. A1978; 1:50,000) or anti-GAPDH (Genesearch, 5174; 1:18,000), and anti-rabbit or anti-mouse HRP-linked secondary antibodies (VWR International, Murarrie, QLD, Australia, Cat. NA9340, NA9310, 1:5000).

### RNA isolation and PCR

RNA was isolated with QIAzol Lysis Reagent (QIAGEN, Chadstone, VIC Australia, Cat. 79306) according to the manufacturer's instructions. cDNA was synthesized from 2 µg of total RNA with the Tetro cDNA Synthesis Kit (Bioline, Alexandria, NSW, Australia, Cat. 65050). PCR was performed with DNA polymerase (Bioline, Cat. 21040). The following primer pairs were used to specifically amplify alpha fetoprotein (*Afp*; Fwd: 5′-TCGTATTCCAACAGGAGG-3′, Rev: 5′-AGGCTTTTGCTTCACCAG-3′), albumin (*Alb*; Fwd: 5′-CTTAAACCGATGGGCGATCTCACT-3′, Rev: 5′-CCCCACTAGCCTCTGGCAAAAT-3′), glyceraldehyde 3-phosphate dehydrogenase (*Gapdh*; Fwd: 5′-TGTTCCTACCCCCAATGTGT-3′, Rev: 5′-TGTGAGGGAGATRGCTCAGTG-3′), hepatocyte nuclear factor homeobox 1B (*Hnf1b*; Fwd: 5′*-*CAGCCAGTCGGTTTTACAGC-3′, Rev: 5′-TCCTCCCGACACTGTGATCT-3′), *Hnf4α* (Fwd: 5′-CAGCAATGGACAGATGTGTGA-3′, Rev: 5′-TGGTGATGGCTGTGGAGTC-3′) and tyrosine aminotransferase (*Tat*; Fwd: 5′-GGGTTGTCTGCCATTCCT-3′, Rev: 5′-TTCTGGGAAGTGCTCCATCT-3′). Primers were synthesized by Geneworks (Hindmarsh, SA, Australia). PCR amplicons were separated on agarose gels and visualized using a ChemiDoc XRS (Bio-Rad). All PCR products were verified by sequencing for authenticity.

### Statistical analyses

Data is presented as the mean±s.e.m. Statistical significance was determined using a Student's *t*-test or one-way ANOVA with Bonferroni's multiple comparison post-test using GraphPad Prism^®^ Ver. 4.0b.
